# Psychological Restoration Can Depend on Stimulus-Source Attribution: A Challenge for the Evolutionary Account?

**DOI:** 10.3389/fpsyg.2016.01831

**Published:** 2016-11-23

**Authors:** Andreas Haga, Niklas Halin, Mattias Holmgren, Patrik Sörqvist

**Affiliations:** Department of Building, Energy and Environmental Engineering, University of GävleGävle, Sweden

**Keywords:** restorative environments, nature environment, built environment, evolutionary account, stimulus-source attribution, psychological restoration

## Abstract

Visiting or viewing nature environments can have restorative psychological effects, while exposure to the built environment typically has less positive effects. A classic view is that this difference in restorative potential of nature and built environments depends on differences in the intrinsic characteristics of the stimuli. In addition, an evolutionary account is often assumed whereby restoration is believed to be a hardwired response to nature’s stimulus-features. Here, we propose the novel hypothesis that the restorative effects of a stimulus do not entirely depend on the stimulus-features *per se*, but also on the meaning that people assign to the stimulus. Participants conducted cognitively demanding tests prior to and after a brief pause. During the pause, the participants were exposed to an ambiguous sound consisting of pink noise with white noise interspersed. Participants in the “nature sound-source condition” were told that the sound originated from a nature scene with a waterfall; participants in the “industrial sound-source condition” were told that the sound originated from an industrial environment with machinery; and participants in the “control condition” were told nothing about the sound origin. Self-reported mental exhaustion showed that participants in the nature sound-source condition were more psychologically restored after the pause than participants in the industrial sound-source condition. One potential interpretation of the results is that restoration from nature experiences depends on learned, positive associations with nature; not only on hardwired responses shaped by evolution.

## Introduction

Natural settings (e.g., forests, lakes and mountains) have greater restorative effects on people than built settings (Ulrich, 1984; Kaplan and Kaplan, 1989; Berman et al., 2008; Hartig et al., 2014) as shown in both field (Hartig et al., 1991) and laboratory studies (Jahncke et al., 2011). The restorative effects of nature have potential to improve health and well-being across a wide range of everyday settings. Restoration with nature exposure is, for example, considered in contemporary public health management (Gulwadi, 2006; Lee et al., 2009; Hartig et al., 2014), urban planning (Van den Berg et al., 2014; Bratman et al., 2015) and indoor environment design (Bringslimark et al., 2009).

From a theoretical viewpoint, a range of studies have been conducted that seek to understand the fundamental mechanisms underpinning the restorative effects of nature and, specifically, what makes nature different from other environments with regard to its restorative potential (e.g., Berto, 2005; Karras et al., 2015; Joye et al., 2016). One view is that people’s positive response to nature stimuli has been shaped by evolution and people are genetically hardwired to respond to nature’s specific stimulus features in healthy ways (Joye and Van den Berg, 2011). This view justifies studies that aim to understand why natural environments are more restorative than their counterparts by investigating how differences in stimulus features underpin psychological restoration. Another view is that learned associations underpin the restorative effects of nature. On this top-down view, a stimulus is not restorative because it maps on to a hardwired response, but because the person who is restored has positive and healthy experiences with the stimulus. Such top-down effects on psychological restoration can, for example, be found in people’s associations with bird sound (Ratcliffe et al., 2016). In the current study, we explore the top-down view of restoration from a novel angle. Here, we assume that the restorative qualities of a stimulus are not entirely attributable to the physical characteristics of the stimulus, but also to learning factors that shape perception of the stimulus and the meaning that is assigned to the stimulus. Specifically, we investigate whether the same stimulus can have different restorative effects depending on which source the stimulus is attributed to.

Many laboratory studies investigate psychological restoration with the use of an experimental setup wherein the participants conduct a mentally fatiguing task prior to, and after, a brief break. During the break, the participants are either exposed to a nature-related visual stimulus or to a visual stimulus from the built environment category (e.g., Devlin and Arneill, 2003; Berto, 2005; Benfield et al., 2014). A general message from these studies is that visiting natural environments—or simply viewing nature scenes—can help people recover faster from mental fatigue (Kaplan, 2001; Hartig et al., 2003; Berto, 2005; Berman et al., 2008; Bratman et al., 2012) and it also contributes to overall happiness (Van den Berg et al., 2003). For example, participants who look at a green rooftop for 40 s during the break appear to be more mentally restored than participants who look at a bare concrete roof, as found both in subjective ratings and an increase in test performance after the break (Lee et al., 2015). It should be noted though, that the restorative effects of nature exposure on subjective ratings appear to be more easily replicated than the effect on cognitive performance (Bergman et al., 2008; Emfield and Neider, 2014).

In this experimental paradigm, it has been found that some specific stimulus-features associated with nature underpin its restorative effects. For example, pictures of natural environments (e.g., trees) are more restorative than pictures of industrial environments, and viewing nature environments is also more restorative than effortlessly viewing geometrical patterns (Berto, 2005). A key stimulus feature that makes nature environments more restorative than other environments could be their fractal structure (Joye and Van den Berg, 2011; Joye et al., 2016; see also Hagerhall et al., 2004). A fractal structure is characterized by repeating patterns when the objects (e.g., trees) are viewed at increasingly fine magnifications, which makes them less effortful and more fascinating than built environments (cf. Kaplan, 1995). Fascination—defined as effortless-interest driven attention (Berto, 2011)—is a key process in restoration according to the attention restoration theory (ART; Kaplan, 1995). Specifically, it is assumed that some stimuli are more restorative than other stimuli, because they have certain features that make people respond with fascination when the stimuli are perceived (Kaplan, 1978).

However, there seems to be more to why a stimulus is restorative than its stimulus features. Auditory stimuli and visual stimuli, for example, can both have restorative effects even though they have different stimulus features. For instance, sound that originates from a nature source (Ratcliffe et al., 2013; Benfield et al., 2014; Emfield and Neider, 2014; Jahncke et al., 2015) has been shown to help restore mental fatigue to a greater extent than exposure to noise (e.g., ventilation noise; Alvarsson et al., 2010) and, similarly, natural sceneries are more restorative than scenes of built environments (Berto, 2005; Lee et al., 2015). Hence, stimuli with clearly different physical characteristics (pictures vs. sound) can have similar restorative effects. The similarity between auditory and visual nature-related stimuli suggests that it is not the stimulus features *per se* that underpins restoration but instead the meaning that is attributed to the stimulus. Specifically, a stimulus appears to be restorative when there are positive associations with the stimulus.

The positive association view of psychological restoration is consistent with studies on perception showing that cognitive, top-down factors can shape how a stimulus is perceived. For instance, people like a smell they believe comes from parmesan cheese, while they dislike the exact same smell if they instead believe it comes from vomit (Herz and von Clef, 2001); and people prefer the taste of a cup of coffee labeled eco-friendly over a conventional labeled alternative, even though the two cups contain identical coffee (Sörqvist et al., 2013). It is hence not only the sensory properties of the stimulus that determine how the stimulus is perceived; it is in part determined by the source attribution of the stimulus (Herz, 2000; Lee et al., 2006), especially if the stimulus is ambiguous (Herz and von Clef, 2001). Similar findings have been reported in the context of sound stimuli. Bergman et al. (2008) showed that a sound is more annoying when the sound is associated with a factory, in comparison with when the same sound is associated with a nature environment. Moreover, people tend to prefer the light from a light source when they believe the light source is environmentally friendly compared to when they do not believe that the light source is environmentally friendly (Sörqvist et al., 2015). Taken together, the meaning that is attributed to a stimulus can change how it is perceived, and perception seems to be shaped by a preference bias for nature (and nature-protecting) sources.

The purpose of the current study was to test whether restorative effects of a stimulus, at least in part, depend on stimulus-source attribution. To this end, the bottom-up (or stimulus driven) part of perception has to be experimentally separated from the top-down (or cognitively driven) part of perception. One way to do this is to use a single stimulus (thereby holding the bottom-up part of perception constant) but to tell the participants in one condition that the stimulus originates from a nature source and the participants in another condition that the stimulus originates from an industrial source (thereby manipulating the top-down part of perception). With this technique, the stimulus features are held constant but the meaning that is attributed to the stimulus are experimentally varied. Most experimental studies exploring the effect of restorative environments have used images as stimuli, but images are not suitable for the top-down manipulation required in the current experiment. With sound, however, it is easier to create a stimulus that is ambiguous enough to be associated with either a source derived from nature or a source from a non-nature environment.

Based on the top-down view of psychological restoration, we hypothesized that participants who were told that the sound originated from a nature environment would perceive the sound as more restorative and pleasant than participants who were told that the sound originated from an industrial environment. We also predicted that participants who were told that the sound had a natural origin would be more psychologically restored after listening to the sound—both in the context of self-reported fatigue and possibly also in the context of a cognitive performance measure. For comparison purposes, a control condition was also included in the experiment with participants who did not receive any information about the sound origin. We predicted that the experienced restoration in the control group would largely depend on how the participants spontaneously classified the sound source. If these predictions are confirmed, the results would support a top-down account of psychological restoration and show that hardwired responses to specific stimulus features alone cannot fully explain why natural stimuli have restorative effects on people.

## Materials and Methods

### Participants

Ninety university students in varied disciplines (68% female) participated in the experiment (mean age = 24.76 years, *SD* = 4.60). In order to recruit participants, flyers were posted around the University, with information that the test was about solving problems at a computer. Each participant was offered a small honorarium for their participation. The study was approved by the Research Ethics Review Board at Uppsala University (Dnr 2015/475).

### Materials

#### Sound

The sound, which was used in all conditions, consisted of a continuous pink noise (sound with the same average power in each octave band; 180 s). Short bursts of white noise (sound with the same average power in each 1-Hz frequency band; 1000 ms) were interspersed at pseudorandom intervals (the white noise was presented every 3rd, 4th, 5th, 6th, or 7th second, *M* = 5 sec). Sound was presented through headphones (Sennheiser 202) at approximately 55 dBA Leq (e.g., office sound standards).

#### The Attention Network Test

A version of the attention network test (ANT) was used to assess cognitive control (Redick and Engle, 2006). All stimuli [which consisted of five “arrows” (e.g.,>>>>>), or one arrow surrounded by dashes (e.g., – >–)] were presented on a computer screen for 1700 ms followed by a pause (i.e., 400 ms) before the next stimuli appeared. There were three types of trials: congruent, incongruent, and neutral. In the congruent trials, all arrows were pointing in the same directions (e.g., <<<<<). In this type of trial, all stimuli match the same response alternative and hence there is no cognitive conflict between the target and the flankers. In the incongruent trials, the middle arrow was pointing in the opposite direction from the surrounding arrows (e.g., <<><<). Hence, in this type of trial the participants would have to resolve a cognitive conflict between the flankers and the targets by inhibiting the inappropriate response that is cued by the flankers. And in the neutral trials, the central arrow was surrounded by dashes (e.g., –<–) which did not match any potential response alternative. The neutral trials were included as a baseline condition. The key measure in this task is the difference in response latency between the congruent and the incongruent trials, which would reflect the cost associated with the need to resolve the cognitive conflict between target and flankers. Participants were asked to indicate the direction of the central arrow by pressing the corresponding arrow keyboard key. They were told to respond as quickly and accurately as possible. The test began with 6 practice trials (two of each type), which were not considered in the analysis. The practice trials were followed by 120 trials (40 of each trial type).

### Design and Procedure

The participants were tested individually in a laboratory room seated in front of a computer. A mixed within-between participants experimental design was used. During the experiment, the participants conducted three ANT tests. Between the first and the second ANT tests, they conducted cognitively demanding tasks (e.g., prose memory test where the procedure is to read texts and then answer questions about the text, and size-comparison span [SIC SPAN] working memory tests) for 40 min. And between the second and the third ANT test, the participants received a brief 3-minute break. The sound was played back during the break. Each participant was randomly allocated to one of three between-participants conditions: one third of the participants (*N* = 30) were told, prior to the 3-min break, that the sound originated from a “nature environment with a streaming waterfall” (the nature sound-source condition), another third (*N* = 30) was told that the sound originated from an “industrial environment with an active machinery” (the industrial sound-source condition), and the last third (*N* = 30) was not told anything about the sound source (the control condition).

### Subjective Ratings

Before the onset of the cognitively fatiguing tasks, just before the break during which the participants listened to the sound, and immediately after the break, participants answered three questions: *“At this moment, how mentally fatigued are you?,” “At this moment, how easy is it for you to concentrate?”*, and *“At this moment, how stressed are you?”*. Responses were made on a scale from 1 to 9 (end-points labeled) by pressing the corresponding number key on the computer keyboard. The responses were inverted for the concentration question. The intercorrelations between the variables were high. Mean mental fatigue was positively correlated to mean concentration, *r*(86) = 0.52, *p* < 0.001, and to stress, *r*(86) = 0.45, *p* < 0.001, as was mean concentration to mean stress, *r*(86) = 0.25, *p* = 0.016. Because of this, the variables were collapsed into an index of mental exhaustion (i.e., the mean value of the three variables, mental fatigue, concentration and stress (Cronbach’s alpha = 0.67), wherein higher values corresponded to more mental exhaustion. After listening to the sound during the 3-minute break, the participants were also asked to respond (on a scale ranging from 1 to 9, end-points labeled) to questions regarding the sound, namely: *“How restorative was the sound?” “How pleasant was the sound?”* and *“How relaxing was the sound?”* They were also given an open-ended question: *“Describe, with one sentence, what you thought about when you listened to the sound?”* The intercorrelations between the questions on the sounds’ restorative qualities were high. There was a positive correlation between estimates of how restorative the sound was and how pleasant the sound was, *r*(86) = 0.55, *p* < 0.001, as between estimate of how restorative and how relaxing the sound was, *r*(86) = 0.69, *p* < 0.001, as between how pleasant and how relaxing the sound was, *r*(86) = 0.77, *p* < 0.001 Thus, these variables were collapsed to create an index of the subjective evaluation of the restorative qualities of the sound (i.e., the mean value of the three variables, restorative, pleasant and relaxing, Cronbach’s alpha = 0.86). Higher values corresponded to the sound having greater restorative effect.

## Results

### Subjective Ratings of the Restorative Effects of the Sound

**FIGURE 1:**
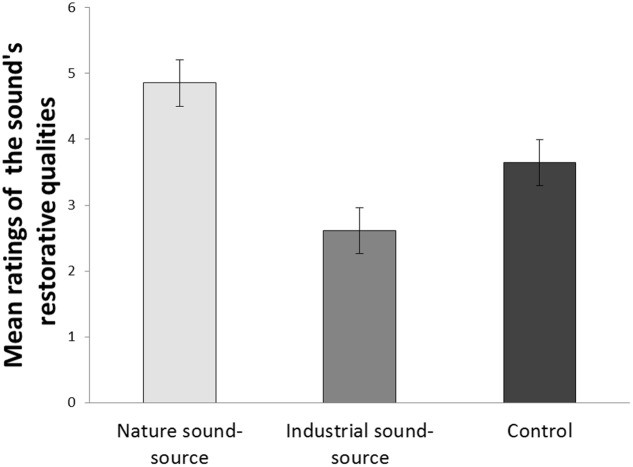
**Mean subjective ratings of the sound’s restorative qualities across the three sound-source conditions.** Error bars represent standard error of means.

As can be seen in **Figure 1**, participants in the “nature sound-source” condition rated the sound as being more restorative compared to the other two conditions. This difference between conditions was significant, as indicated by a univariate ANOVA, *F*(2, 87) = 10.25, *p* < 0.001, $\eta_{p}^{2}$ = 0.19. A Tukey HSD *post hoc* test showed that the mean difference between the “nature sound-source” condition and the “industrial sound-source” condition was significant (*M*_diff_ = 2.24; *p* < 0.001), as well as the difference between the “nature sound-source” condition and the control condition (*M*_diff_ = 1.21; *p* = 0.044). The control condition did not differ significantly from the “industrial sound-source” condition (*M*_diff_ = 1.03; *p* = 0.099).

To further explore the role of source attribution in determining the sound’s restorative effects, the 30 participants in the control condition were grouped according to their open-ended responses to the question about what they were thinking of when they listened to the sound. The “nature-related sound” group (*N* = 12) consisted of participants who had been thinking about something nature related (e.g., a breeze across the ocean), and the “non-nature related sound” group (*N* = 15) consisted of participants who had been thinking about something artificial or otherwise unrelated to nature (e.g., a poor radio broadcast). A group of 3 participants were excluded from this analysis because it was impossible to classify them into the nature-related or non-nature related group based on what they said they had been thinking about while listening to the sound (e.g., “what I will eat for lunch”). Participants in the “nature-related sound” group rated the sound as more restorative (*M* = 5.28, *SD* = 2.02) compared to those in the “non-nature related sound” group (*M* = 2.31, *SD* = 1.17), and this difference was significant as shown by an independent-samples *t*-test, *t*(25) = 4.79, *p* < 0.001, Cohen’s *d* = 1.80.

### Self-Reports of Mental Exhaustion

**FIGURE 2:**
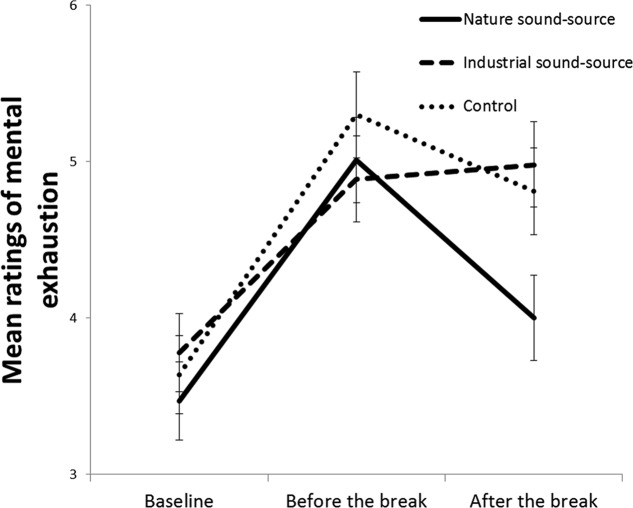
**Mean ratings of the mental exhaustion index across the three times of data collection for the three sound-source conditions.** Error bars represent standard error of means.

We checked on the degree to which participants in the three sound-source conditions differed from each other in self-reported mental fatigue before the experiment started. The results of a univariate ANOVA showed that participants in each sound-source condition had reported similar levels of mental fatigue at baseline, *F*(2,87) = 0.38, *p* = 0.684, $\eta_{p}^{2}$ = 0.001. As presented in **Figure 2**, mental exhaustion increased between baseline and the data collection before the break in all three conditions. After the break, participants in the “nature-sound source” condition reported being less mentally exhausted than participants in the “industry-sound source” condition. This conclusion was confirmed by a 3 (Sound-source condition: nature vs. industry vs. control) × 3 (Time of data collection: baseline vs. before the break vs. after the break) mixed ANOVA that revealed a main effect of time of data collection, *F*(2, 174) = 49.54, *p* < 0.001, $\eta_{p}^{2}$ = 0.36, but not of sound-source condition, *F*(2, 87) = 1.01, *p* = 0.337, $\eta_{p}^{2}$ = 0.03, and a significant interaction between the two factors, *F*(4, 174) = 2.70, *p* = 0.032, $\eta_{p}^{2}$ = 0.06. Follow-up paired-samples *t*-tests showed that participants in the “nature sound-source” condition were less mentally exhausted after the break compared to before the break, *M*_diff_ = 1.01, *t*(29) = 5.32, *p* < 0.001, whilst the difference between before and after the break was not significant for the “industrial sound-source” condition, *M*_diff_ = –0.09, *t*(29) = –0.50, *p* = 0.621, nor in the control condition, *M*_diff_ = 0.49; *t*(29) = 1.56, *p* = 0.129.

**FIGURE 3:**
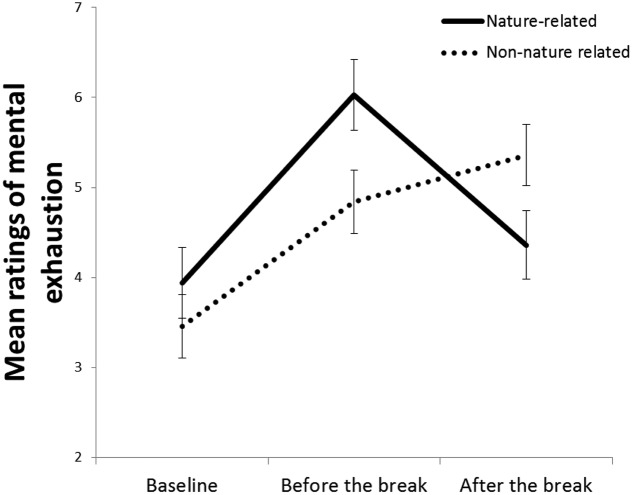
**Mean ratings of the mental exhaustion index across the three times of data collection for participants in the control condition who spontaneously attributed the sound to a nature-related source and those in the same condition who attributed the sound to a non-nature related source.** Error bars represent standard error of means.

Additional analyses with participants from the control condition were conducted separately to investigate whether participants’ spontaneous sound source attribution (as seen in the descriptions of what they had been thinking about when they listened to the sound) influenced the effects of the sound on the participants’ subjective mental exhaustion. Participants in the “nature-related sound” group reported slightly higher score on the mental exhaustion index at baseline (*M* = 3.94, *SD* = 1.74) compared to participants in the “non-nature related” sound (*M* = 3.46, *SD* = 0.97), but an independent samples *t*-test showed that this difference was not significant, *t*(25) = 0.91, *p* = 0.370. As can been seen in **Figure 3**, both groups’ score on the mental exhaustion index increased during the period between baseline and before the break, but participants in the “nature-related sound” group reported lower mental exhaustion after the break, while participants in the “non-nature related sound” group reported higher mental exhaustion after the break. This conclusion was supported by a 2 (Source attribution group: nature-related sound source vs. non-nature related sound source) × 3 (Time of data collection: baseline vs. before the break vs. after the break) mixed ANOVA, with source attribution group as between-participants factor and time of data collection as within-participants factor. The analysis revealed a main effect of time of data collection, *F*(2, 50) = 20.68, *p* < 0.001, $\eta_{p}^{2}$ = 0.45, but not of source attribution group, *F*(1, 25) = 0.29, *p* = 0.597, $\eta_{p}^{2}$ = 0.01, and a significant interaction between the two factors, *F*(2, 50) = 8.21, *p* = 0.001, $\eta_{p}^{2}$ = 0.25. Follow-up paired-sample *t*-tests showed that participants in the “nature-related sound” group were less mentally exhausted after the break compared to before the break, *M*_diff_ = 1.67, *t*(11) = 4.02, *p* = 0.002, but the difference between these two moments was not significant for the “non-nature related sound” group, *M*_diff_ = –0.51, *t*(14) = –1.47, *p* = 0.163.

**Table 1:** Means (and standard deviations) for reaction time data (milliseconds) on the three types of trials in the attention network test (ANT), across the three sound-source conditions and the three times of data collection (1 = baseline, 2 = before the break, 3 = after the break).

	Nature sound-source condition			Industrial sound-source condition			Control condition		
Type of trial	1	2	3	1	2	3	1	2	3
Incongruent trial	563.36 (63.64)	523.23 (46.52)	516.49 (48.52)	539.31 (90.64)	498.13 (55.96)	491.11 (54.99)	570.53 (95.17)	520.71 (63.74)	503.58 (57.15)
Congruent trial	485.67 (63.72)	439.14 (36.19)	434.23 (38.71)	450.32 (65.29)	422.96 (41.44)	423.02 (44.78)	492.33 (85.81)	451.70 (55.60)	430.69 (49.54)
Neutral trial	473.02 (52.71)	438.89 (36.31)	429.73 (37.67)	443.85 (51.55)	416.38 (38.80)	418.72 (37.60)	491.59 (85.36)	450.97 (53.62)	431.81 (47.19)

### ANT Performance

All response-time mean values for the three types of trials in the ANT test across the three sound-source conditions are presented in **Table 1**. Of particular interest for the current study was the interaction between sound-source condition and type of trial on response latencies, because this interaction would reveal differences in cognitive control as a result of the sound-source manipulation. However, a 3 (Sound-source condition: nature vs. industry vs. control) × 3 (Time of data collection: baseline vs. before the break vs. after the break) × 3 (Type of trial: incongruent vs. congruent vs. neutral) the mixed ANOVA did not find any significant main effect of sound-source condition or any type of interaction wherein sound-source condition was involved, either when data were separated on time of data collection or when the data were collapsed across time of data collection. There was no statistical difference between the “nature sound-source” condition and the “industrial sound-source” condition, nor between the “nature-related sound” group and the “non-nature related sound” group in the control condition. The ANT test is designed to measure response time, not the accuracy of the responses, since high accuracy can easily be achieved simply by using much time for each response. Because of this very high accuracy is expected in all trial types, the response time analyses are the only performance-related analyses of relevance here. We nonetheless conducted analyses with accuracy as dependent variable. These revealed no significant effects of the experimental manipulation.

## Discussion

The aim of this study was to examine whether the source to which a stimulus is attributed influences the restorative effects of the stimulus. All participants listened to an ambiguous sound during a brief break from cognitive work, and those participants who were told that the sound originated from a nature source reported being less mental exhausted after the break in comparison with participants who were told that the sound originated from an industrial source, although the sound was identical. Furthermore, participants in a control condition—who were not told anything about the origin of the sound—responded differently to the sound depending on which source they spontaneously attributed the sound to. Participants who described the sound as nature-related perceived it as being more restorative, and also felt less mentally exhausted after listening to the sound, compared to participants who had been thinking about something non-nature related when listening to the sound.

The experimental technique used here made it possible to separate bottom-up processes of perception—which depend on stimulus characteristics and features—and top-down processes of perception—which depend on cognitive factors like expectations and memory. Here, the bottom-up processes were held constant—as the stimulus features were the same in both (nature sound-source and industrial sound-source) experimental conditions—while the top-down processes were manipulated through instructions. Because of this, the differences in the restorative effects of the sound between the experimental conditions must be attributed to differences in top-down processes that shaped how the sound was perceived.

The strongest theoretical implication of the current findings is that stimulus-source attribution and the meaning assigned to a stimulus influences the stimulus’ potential for supporting psychological restoration. Differences in stimulus features—such as fractal structures—may also contribute to differences in stimuli’s restorative potential (Joye and Van den Berg, 2011; Joye et al., 2016), but the results reported in the current study suggest that stimulus differences alone cannot fully explain why some stimuli are more restorative than others, at least not when the stimulus is ambiguous. For example, the consistent finding that views of natural environments help people restore from mental fatigue (Berto, 2005; Berman et al., 2008; Lee et al., 2015), at least when compared to views of built environments, can hardly be fully explained in terms of the physical difference (e.g., shapes, colors, architecture) between the two environments—the cognitive components, experiences and associations with nature also contributes to the difference. One such cognitive component could be fascination, as it has been shown that a stimulus’ restorative potential depends on fascination of the stimuli (Berto et al., 2010), or it could be some other cognitive component that mediates the effect of the stimulus on the restoration outcome. The point to be made here is that these cognitive (top-down) components of restoration are not necessarily driven by stimulus features. It seems as if the restorative potential in nature does not depend on hardwired responses to nature’s specific stimulus features that have been shaped by evolution, at least not entirely.

The top-down view outline here, that learned experiences and positive associations underpin restoration, rather than a hardwired preference bias for nature environments, is in line with the view that bird sound is perceived as more restorative when it originates from birds who behave in ways people appreciate (Ratcliffe et al., 2016). The top-down view is furthermore in line with the finding that built settings can also have healthy effects on people (Staats et al., 2016). Whether restorative qualities are attached to a stimulus may well depend on whether the stimulus is attributed to a source to which the perceiver has positive, affective associations, and that is not necessarily a nature source. Nature environments are thereby not categorically good for psychological restoration and built environments are not categorically bad for psychological restoration. For example, Scopelliti and Giuliani (2004) found that natural and built environments can have similar restorative potentials and whether they do depends on the environments’ social and affective dimensions. An interesting agenda for future research would be to expand on existing research (for a review, see Korpela and Staats, 2014) into how social (e.g., other people’s presence; Staats and Hartig, 2004), affective (e.g., place attachment) and cognitive factors (top-down factors like source attribution) each contribute to the restorative experience of an environment, both within the context of natural environments and built environments.

It should be mentioned, though, that one way in which an evolutionary account could encompass the results from the current study, is by assuming that evolution has not shaped hardwired responses to nature’s stimulus features, but rather hardwired responses to any stimulus that is perceived as originating from nature. Similarly, positive associations with bird sound from friendly birds could also be hardwired rather than learned, under the assumption that evolution has shaped a preference bias for harmless birds and other harmless animals. Therefore, the most reliable claim that can be made from the current study is that an evolutionary account, by which restoration depends entirely on hardwired responses to specific stimulus features, is wrong.

On a methodological note, it should be noted that the sound stimulus used in the current study was ambiguous and did in fact not have a nature sound source. The reason why the sound was ambiguous was to make the sound-source instruction manipulation possible. However, this sound selection also limits the generalizability of the results. The role of top-down factors in the restoration process may be different when people encounter non-ambiguous nature stimuli in their everyday life. Another methodological concern of the current study is the potential role of demand characteristics (the participants’ view of what the researcher is hoping to find). The results from studies on the restorative effects of nature may be influenced by potential demand characteristics as well as political and moral obligations (e.g., a feeling of obligation amongst the participants to try to demonstrate the benefits of nature). There is reason to doubt that the results of the current experiment are within the reach of a demand characteristics explanation, however. One argument which speaks against the demand characteristics explanation is the fact that the effects of the experimental manipulation were selective. If demand characteristics were responsible for the difference between conditions, a difference between conditions should also have been expressed in the context of the performance measure (the ANT) in the same way as in the context of the subjective ratings, but this was not the case. Moreover, the results in the control condition also speak against the demand characteristics explanation, because the participants in the control condition were not informed about the sound source. Arguably, they did not act on beliefs about what the researcher was hoping to find, but still they behaved in patterns similar to the participants in the other two experimental conditions (when split into a group who attributed the sound to a nature source and a group who attributed the sound to a non-nature source).

## Conclusion

Research should start considering that in the psychological restoration process bottom-up and top-down processes may be separable in some experimental contexts, at least when the stimuli are ambiguous. Though more research is needed, the current study may suggest that a strong evolutionary perspective, by which restoration depends on hardwired responses to nature’s specific stimulus features, is wrong. Top-down cognitive components, possibly in the shape of associations people have learned from experiences with various environments, seem to influence the restorative effects of a stimulus.

## Author Contributions

AH, NH, MH, and PS conceived the study. AH and NH collected data. AH and NH analyzed the results. AH, NH, MH, and PS wrote the paper.

## Conflict of Interest Statement

The authors declare that the research was conducted in the absence of any commercial or financial relationships that could be construed as a potential conflict of interest.
